# Multifaced Roles of the αvβ3 Integrin in Ehlers–Danlos and Arterial Tortuosity Syndromes’ Dermal Fibroblasts

**DOI:** 10.3390/ijms19040982

**Published:** 2018-03-26

**Authors:** Nicoletta Zoppi, Nicola Chiarelli, Marco Ritelli, Marina Colombi

**Affiliations:** Division of Biology and Genetics, Department of Molecular and Translational Medicine, School of Medicine, University of Brescia, 25123 Brescia, Italy; nicoletta.zoppi@unibs.it (N.Z.); nicola.chiarelli@unibs.it (N.C.); marco.ritelli@unibs.it (M.R.)

**Keywords:** αvβ3 integrin, extracellular matrix, fibronectin, Ehlers–Danlos syndromes, arterial tortuosity syndrome, apoptosis, fibroblast-to-myofibroblast transition

## Abstract

The αvβ3 integrin, an endothelial cells’ receptor-binding fibronectin (FN) in the extracellular matrix (ECM) of blood vessels, regulates ECM remodeling during migration, invasion, angiogenesis, wound healing and inflammation, and is also involved in the epithelial mesenchymal transition. In vitro-grown human control fibroblasts organize a fibrillar network of FN, which is preferentially bound on the entire cell surface to its canonical α5β1 integrin receptor, whereas the αvβ3 integrin is present only in rare patches in focal contacts. We report on the preferential recruitment of the αvβ3 integrin, due to the lack of FN–ECM and its canonical integrin receptor, in dermal fibroblasts from Ehlers–Danlos syndromes (EDS) and arterial tortuosity syndrome (ATS), which are rare multisystem connective tissue disorders. We review our previous findings that unraveled different biological mechanisms elicited by the αvβ3 integrin in fibroblasts derived from patients affected with classical (cEDS), vascular (vEDS), hypermobile EDS (hEDS), hypermobility spectrum disorders (HSD), and ATS. In cEDS and vEDS, respectively, due to defective type V and type III collagens, αvβ3 rescues patients’ fibroblasts from *anoikis* through a paxillin-p60Src-mediated cross-talk with the EGF receptor. In hEDS and HSD, without a defined molecular basis, the αvβ3 integrin transduces to the ILK-Snail1-axis inducing a fibroblast-to-myofibroblast-transition. In ATS cells, the deficiency of the dehydroascorbic acid transporter GLUT10 leads to redox imbalance, ECM disarray together with the activation of a non-canonical αvβ3 integrin-TGFBRII signaling, involving p125FAK/p60Src/p38MAPK. The characterization of these different biological functions triggered by αvβ3 provides insights into the multifaced nature of this integrin, at least in cultured dermal fibroblasts, offering future perspectives for research in this field.

## 1. Introduction

### 1.1. Extracellular Matrix 

Connective tissues are a mixture of different cell types and protein components organized in the reticular network of the extracellular matrix (ECM), which ensures tissue differentiation, structure, integrity and elasticity. ECM regulates many physiological cells’ activities, including adhesion, proliferation, survival, migration and apoptosis and it is also involved in phenotypic transition during morphogenesis and wound healing [1,2,3,4]. The ECM in vivo provides the interstitial matrix of tissues or, in basement membranes, pericellular layers binding the parenchymal cells to maintain them closely anchored to the connective tissue. The ECM is a dynamic structure undergoing organization and remodeling where ECM components are deposited, degraded or modified [5,6]. This constant turnover is essential during development and to reorganize the tissue architecture in physiological and pathological conditions [7,8]. The ECM composition is specific for different tissues and their physical properties, and about 300 macromolecules are defined to form the so-called matrisome, including collagens (COLLs), fibronectin (FN), elastin (ELN), ELN-associated proteins, i.e., fibrillins (FBNs), fibulins, and EMILINs, tenascins, laminins, osteonectin, osteopontin, glycosaminoglycans and proteoglycans (PGs) [9,10,11]. These constituents associate with each other to form a hydrated three-dimensional network that is also a reservoir of bioactive molecules and growth factors, such as the epidermal growth factor (EGF) and the transforming growth factor β (TGF-β), which regulate different cells’ activities [1]. The ECM is the result of biosynthetic activity and proteolytic degradation by cells that produce either structural ECM molecules or proteases, such as metalloproteases (MMPs), disintegrin and metalloproteinases, and plasminogen activators [12,13,14,15,16,17]. The unbalance between these two activities elicits an abnormal ECM organization, as observed in stiffness and fibrosis [5,8,18,19,20], or ECM degradation and loss occurring in invasive cancer disease [5,8,21,22] and in several heritable connective tissue disorders (HCTDs) [5,8,9,23,24,25]. Among the numerous ECM structural components, the FN is a high molecular weight (470–500 kDa) heterodimeric glycoprotein that is mainly expressed during embryogenesis and wound healing in a fibrillar form, whereas it is present in a globular form in the bloodstream [26,27,28,29]. The fibrillar FN is encoded by mRNA that contains the EDA segment (EDA^+^FN), whereas the circulating FN does not (EDA^–^FN) [30,31,32,33,34]. Other internal regions derived from alternative splicing at two additional sites i.e., EDB and IIICS [35], are known to play a role in tissue differentiation and are modulated in different pathological conditions [31,34,36,37,38,39,40,41].

### 1.2. Integrins

ECM proteins are connected with the cell cytoskeleton by integrins, a family of 24 heterodimeric transmembrane receptors containing an α and a β subunit, which transduce mechanical forces derived from the ECM into the cell or forces generated by the cytoskeleton, i.e., actin microfilaments, to the extracellular environment. The main integrins’ functions are reviewed elsewhere [42,43,44,45,46,47]. Integrin-mediated mechanotransduction induces cellular responses that drive development, cell movement, proliferation, survival and tissue homeostasis [48]. The binding of integrins to the cytoskeleton orchestrates cell migration [49], cell-ECM interaction, a survival signal that inhibit pro-apoptotic proteins [50,51], and an appropriate ECM releases several growth factors driving the cell cycle [50,52]. Cell-matrix adhesions are integrin-mediated molecular sites where the mechanical cues are converted into biochemical signaling. Different adhesive structures exist, such as nascent, focal, and fibrillar adhesions, which vary in shape, subcellular location, lifetime, functions and protein composition. Movements and interactions of their scaffold and signaling protein components lead to the assembly/disassembly, maturation, and interconversion of these dynamic architectures both in vivo and in vitro [53,54,55]. In cultured fibroblasts, the ECM adhesions can be classified in two major types, recruiting different settings of integrins and cytoskeleton anchor molecules, i.e., focal and fibrillar adhesions. Focal adhesions are transmembrane anchorage sites located at the fibroblast periphery between cells and underlying ECM fibrils. They are associated with the end of stress fibers and usually contain the αvβ3 integrin interacting with a complex pattern of proteins that include vinculin, talin, paxillin, α-actinin, zyxin, p125 focal-adhesion kinase (p125FAK), integrin-linked kinase (ILK), and other phosphotyrosine proteins and kinases [53,56,57]. Fibrillar adhesions, arising from focal adhesions, are elongated structures distributed more centrally on the cell surface, enriched in α5β1 integrins bound to tensin, and directly involved in the FN–ECM organization [53,56,58]. 

### 1.3. The αvβ3 Integrin

The αvβ3 integrin, originally named vitronectin receptor [59] because of its predominant ECM ligand, is one of the most promiscuous receptors binding a plethora of at least 21 different ECM proteins, including FN, FBNs, osteopontin, laminin, fibrinogen, von Willebrand factor, thrombospondin, and thrombin [60]. This integrin is widely expressed in endothelial cells (EC) where it is involved in angiogenesis [61,62,63,64], in smooth muscle cells (SMC) [65], myofibroblasts [66,67], osteoclasts, and blood cells, such as monocytes and platelets [59,60]. The recruitment on the cell surface of the αvβ3 integrin is a hallmark of myofibroblasts, specialized cells with both fibroblasts’ and SMCs’ phenotypic characteristics. These cells are activated by inflammatory cytokines and are involved in wound healing [68] and in pathological conditions, such as fibrosis [69] and chronic inflammation [70]. Table 1 summarizes the αvβ3 integrin cell expression, its major ligands and main functions.

As with all integrins, αvβ3 acts as a bidirectional signaling molecule. During “inside-out” signaling, the short cytoplasmic tail of the β3 subunit, through the binding to talin and kindlin, links the integrin to the actin cytoskeleton and elicits conformational changes, e.g., disruption of the intracellular bridges between the cytoplasmic subunits, dissociation of the transmembrane helices, and reorganization of the integrin in a high-affinity binding form that increase the affinity of αvβ3 for the extracellular ligands [71,72,73]. The binding of αvβ3 to the ECM drives the “outside-in” signals by clustering at the plasma membrane of other heterodimers, increase of adhesiveness, and downstream phosphorylation of several kinases for signal transduction. The cytoplasmic domain does not contain intrinsic tyrosine kinase activity and therefore “outside-in” signaling occurs primarily via the recruitment of intracellular signaling kinases, e.g., p125FAK, ILK, Src family kinases, paxillin, and vinculin that are important also for the actin cytoskeleton assembly [74,75]. A selected group of integrins including αvβ3 can stimulate the activation of Ras via interactions of the αv subunits with the adaptor molecule Shc and its association with Grb2 and Sos [76]. These interactions are crucial in adhesion-dependent cell proliferation and survival, as demonstrated by the up-regulation of the antiapoptotic protein Bcl-2 [77]. In addition, the integrin-mediated cell anchorage suppresses the p53 activity in the regulation of apoptosis [78,79]. Besides, the phosphorylation of the mitogen-activated protein kinases (MAPK), phosphoinositide kinase (PI3K)/Akt, and extracellular signal regulated kinase (ERK) is a downstream effect of the αvβ3 integrin activation that regulates cell proliferation, migration/invasion, and cell survival [80]. 

The αvβ3 integrin’s signaling can act synergistically with several growth factor receptors, such as the EGF receptor (EGFR) [81,82], and the TGF-β receptor (TGFBR) [83], also through the cross-talk with their downstream pathways [84,85,86]. For instance, previous studies have shown a direct interaction between αvβ3 and TGFBRII upon stimulation with active TGF-β [83,87].

### 1.4. Heritable Connective Tissue Disorders (HCTDs)

HCTDs comprise a wide range of pleiotropic multisystem diseases mainly affecting the connective tissue of various organ systems, including heart, blood vessels, bone, eyes, skin, joints and lungs. HCTDs result from genetic defects that perturb ECM assembly, maintenance, and homeostasis. Defects in the amount or structure of one of the numerous ECM constituents affect the proper organization and structural integrity of the supporting connective tissues and cause the weakness of bones, skin or vascular tissue which characterizes the disease phenotypes of different HCTDs [88]. Indeed, disease-causing mutations in several ECM-related genes or enzymes involved in biosynthesis or processing of ECM proteins, cause a myriad of HCTDs, e.g., Ehlers–Danlos syndromes (EDS), Osteogenesis imperfecta (OI), Marfan syndrome (MFS), Loeys–Dietz syndromes (LDS), arterial tortuosity syndrome (ATS), and numerous skeletal dysplasias [9]. Many of these disorders show some clinical overlap regarding cardiovascular, skeletal, craniofacial, ocular, and cutaneous features reflecting the common denominator of the ECM perturbation [89].

### 1.5. The Ehlers–Danlos Syndromes (EDS) and Arterial Tortuosity Syndome (ATS)

Among HCTDs, EDS share a variable combination of skin hyperextensibility, joint hypermobility (JHM), and manifestations of generalized connective tissue fragility. The revised 2017 EDS nosology distinguishes 13 different EDS types with 19 causative genes known to date (Table 2) [90]. For a comprehensive clinical and molecular description of all EDS types see the paper by Malfait and coworkers [90]. 

The classical (cEDS), vascular (vEDS) and hypermobile (hEDS) EDS forms account for more than 90% of EDS patients. Briefly, cEDS is characterized by abnormal skin involvement and generalized JHM (gJHM) [90,91], and is mainly caused by mutations in the *COL5A1* and *COL5A2* genes encoding the type V collagen (COLLV) [92,93]. vEDS is characterized by a clinical history of arterial rupture, dissection or aneurysm, rupture of the large intestine, and pregnancy complications at young ages [94]. vEDS is caused by mutations in the *COL3A1* gene that encodes the type III collagen (COLLIII), which is the major expressed collagen in blood vessels and hollow organs [95]. The clinical criteria according to the revised 2017 EDS nosology suggestive for cEDS and vEDS are shown in Table 3 and Table 4. Confirmatory molecular testing is needed to reach a final diagnosis.

hEDS follows an autosomal dominant inheritance pattern with an unknown molecular basis and is mainly characterized by gJHM, joint instability complications, and minor skin changes [90]. The clinical criteria for a hEDS diagnosis according to the revised 2017 EDS nosology are summarized in Table 5. The phenotypic spectrum of hEDS also includes multiple associated symptoms shared with chronic inflammatory systemic diseases. Many of these features are not sufficiently specific nor sensitive to be included in the formal diagnostic criteria. These include, but are not limited to, sleep disturbance, fatigue, postural orthostatic tachycardia, functional gastrointestinal disorders, dysautonomia, anxiety, and depression [96]. Following the new classification, the term hypermobility spectrum disorders (HSD) is an alternative label for patients with symptomatic JHM who do not meet the new criteria for hEDS [97].

EDS are characterized by huge genetic heterogeneity, wide phenotypic variability between the different forms, and clinical overlap with other HCTDs. Indeed, EDS share with other HCTDs such as ATS, MFS, LDS, and OI, some degree of phenotypical overlap of cardiovascular, cutaneous, and skeletal features. Briefly, ATS is characterized by tortuosity and elongation of large- and medium-sized arteries and is caused by loss-of-function mutations in the *SLC2A10* gene encoding the facilitative glucose transporter 10 (GLUT10), which facilitates the uptake of glucose and dehydroascorbic acid (DHA) [98,99]. For an overview of the phenotypic presentation in ATS patients see Table 6. MFS, caused by heterozygous mutations in *FBN1* encoding the ECM protein fibrillin 1, is characterized by cardiovascular, ocular, and skeletal manifestations. The most common cardiovascular phenotype involves aortic aneurysm and dissection at the sinuses of Valsalva [89]. LDS, caused by mutations in different components of the TGF-β signaling pathway, i.e., *TGFBR1, TGFBR2*, *SMAD2*, *SMAD3*, *TGFB2* and *TGFB3*, is mainly characterized by a clinical triad including hypertelorism, bifid uvula or cleft palate, and aortic aneurysm with arterial tortuosity [89]. OI comprises a heterogeneous group of diseases characterized by susceptibility to bone fractures with variable severity. OI display different modes of inheritance with autosomal dominant as the predominant inheritance pattern caused by mutations in the *COL1A1* and *COL1A2* genes (in about 85% of individuals) encoding the α1 and α2 chains of type I COLL (COLLI), respectively [100,101].

### 1.6. Organization of Fibronectin (FN) and Collagens (COLLs) and their Canonical Integrin Receptors in Dermal Fibroblasts from Different EDS Types and Other HCTDs

The skin is one of the connective tissues affected in all EDS types as well as in other HCTDs, and dermal fibroblasts have been shown to represent an excellent in vitro cell model to study ECM organization and molecular mechanisms involved in the pathophysiology of several HCTDs [102,103,104,105,106,107,108,109,110,111,112,113]. In vitro grown human dermal fibroblasts synthesize and secrete several ECM structural proteins that are deposited on the substrate and organized in a network covering the cell layer. In particular, three days after seeding, control fibroblasts organize the ECM of FN (FN–ECM), with a predominant deposition of the EDA^+^FN variant, COLLIII, COLLV, and rare fibrils of COLLI ([102,103,104,105,106,107,108,109,110,111,112,113], and Table 7). The main COLLs receptor expressed by control cells is the α2β1 integrin, whereas FN is preferentially bound to the α5β1 integrin [102,103,104]. In vitro cultured dermal fibroblasts derived from patients affected with different EDSs, except spEDS-*B3GALT6* [109], exhibit a common cellular phenotype that is characterized by rare FN–ECM fibrils in association with reduced/absent patches in the plasma membrane of the canonical FN receptor the α5β1 integrin., Consequently, these FN–ECM-deficient cells show the preferential expression of the alternative FN receptor, i.e., the αvβ3 integrin, which is organized in linear patches both in fibrillar and focal adhesions ([102,103,106,107,110,111,112,113], and Table 7). This phenotype is also observed in dermal fibroblasts from patients with ATS but not in LDS-*TGFBR1*, MFS, and OI cells, which organize an abundant FN–ECM and express the α5β1 integrin and not αvβ3 ([105,114], and Table 7). EDS fibroblasts also show a reduced/absent deposition into the ECM of COLLI and COLLIII, in association with a variable organization of COLLV ([102,103,104,106,107,108,109,110,111,112,113], and Table 7). A similar disorganization of the COLLs–ECM is also observed in LDS-*TGFBR1*, OI, and ATS cells but not in MFS fibroblasts ([105], and Table 7). The abnormal COLLs–ECM depositions observed in all EDS types as well as in OI and ATS cells is associated with the loss of the canonical COLLs α2β1 integrin receptor’s expression ([102,103,106,107,108,109,110,111,112,113], and Table 7). For the genetically defined forms of EDS as well as for OI, the abnormal COLLs–ECM organization and the consequent loss of the α2β1 integrin is easily explained by the underlying molecular defects that include not only anomalies of the collagen primary structure (cEDS, vEDS, and OI), collagen processing (dEDS, aEDS), folding and cross-linking (kEDS), but also defects in glycosaminoglycan biosynthesis (spEDS, mcEDS) that are known to impact COLLs fibril formation and deposition [9]. Concerning hEDS and HSD cells, the lack of COLLs–ECM [107] could partly be explained by the high levels of the active form of the MMP-9 collagenase recently reported in their culture media [106]. In ATS cells, the lack of GLUT10 was shown to affect both the redox homeostasis and the proper processing and secretion of several ECM components, thus accounting for their defective COLLs–ECM [99,105,115,116].

**Figure 1:**
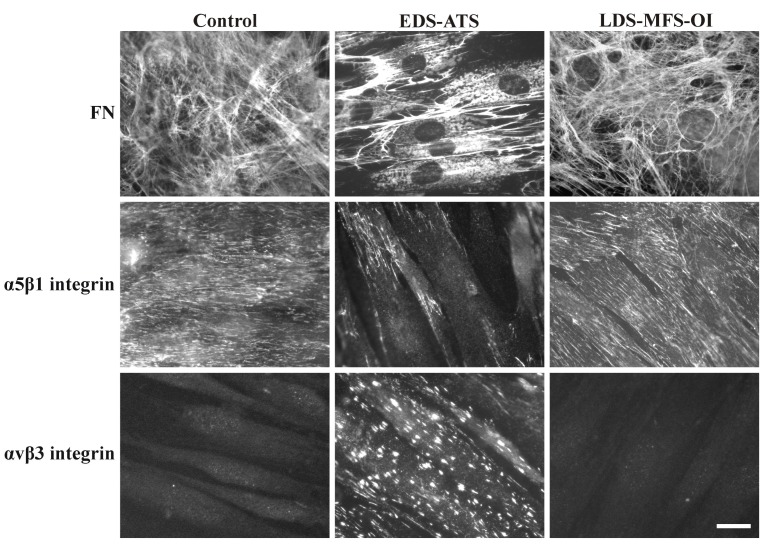
Organization of FN–ECM and expression of the α5β1 and αvβ3 integrin receptors in control and HCTDs dermal fibroblasts. Among the analyzed HCTDs (Table 7), all EDS fibroblasts, except for spEDS-*B3GALT6*, and ATS cells show the FN–ECM disarray, the reduced expression of the canonical FN integrin receptor α5β1 and the consequent recruitment of the αvβ3 integrin. This peculiar phenotype is not observed in cells derived from MFS, LDS, and OI patients. Dermal fibroblasts were immunoreacted with antibodies against FN, α5β1, and αvβ3 integrins as previously described [102,106,107]. Scale bar: 10 μm.

Taken together, the FN–ECM disarray in association with reduced expression of its canonical integrin receptor α5β1 and consequent recruitment of the αvβ3 integrin seems to represent a peculiar in vitro phenotype of the majority of EDS cells as well as ATS fibroblasts (Figure 1). 

Herein, we review our previously reported findings that unraveled different biological mechanisms elicited by the αvβ3 integrin in cEDS, vEDS, hEDS, HSD, and ATS dermal fibroblasts.

## 2. The Pro-Survival Role of the αvβ3 Integrin in Classical EDS (cEDS) and Vascular EDS (vEDS) Dermal Fibroblasts 

In cEDS and vEDS fibroblasts the αvβ3 integrin is abundantly clustered both in focal and fibrillar contact sites, where it drives adhesion either to uncoated or purified FN-coated substrates [102,103]. The functional blocking of this integrin receptor with inhibiting antibodies reduces their adhesive potential corroborating that the αvβ3 integrin is the main adhesive ECM receptor in these EDS fibroblasts. In these cells, the αvβ3 integrin is a FN-binding receptor that sustains the FN assembly, since EDS cells grown in the presence of exogenous purified human plasma FN are induced to organize a FN fibrillar matrix without recruiting the canonical FN receptor α5β1 integrin [103]. This is consistent with the well-known capability of the αvβ3 integrin to bind and assemble a FN–ECM [117].

Moreover, the expression of the αvβ3 integrin in cEDS and vEDS fibroblasts is a downstream effect of COLLV and COLLIII deficiency, respectively, which, in turn, also affects the synthesis of the EDA^–^FN, and its secretion and organization into the ECM [104]. Indeed, the treatment of cEDS and vEDS fibroblasts with purified COLLV and COLLIII respectively restores the COLLs–ECM assembly, induces the up-regulation of the EDA^+^FN expression in association with its organization in a control-like ECM. This COLLs-mediated ECM rescue is associated with the restoration of a canonical cells’ integrin setting, since the αvβ3 integrin patches disappear and both the lacking COLLs receptor α2β1 integrin and the FN-specific α5β1 integrin are organized on the cell surface [102,104]. In cEDS and vEDS fibroblasts, the αvβ3 integrin is activated, as demonstrated by its tyrosine phosphorylation, and it transduces adhesion signals [103]. The adhesion-dependent fibroblasts’ survival is known to be regulated by ECM assembly and turn over. The lack of cell adhesion to the ECM induces the fibroblasts’ growth arrest and apoptosis/*anoikis* [118,119,120,121,122,123,124,125]. ECM-deficient cEDS and vEDS fibroblasts proliferate in vitro as well as control fibroblasts. However, the inhibition of the αvβ3 integrin induces cEDS and vEDS fibroblasts to undergo *anoikis*, suggesting that this receptor plays a key survival role in the rescue of these cells from *anoikis* activated by the ECM disassembly [103]. The survival function of the αvβ3 integrin has been reported in other cell types, i.e., endothelial [126] and tumor [127] cells. In cEDS and vEDS fibroblasts, the anti-apoptotic transduction pathway activated by the αvβ3 integrin does not involve p125FAK [103], which is known to have a central role in the PKB/Akt-mediated activation of the cell cycle and the inhibition of pro-apoptotic mediators, such as Bad and caspases [128,129]. In cEDS and vEDS cells, the down-regulation of p125FAK should explain the low synthesis of the survival protein Bcl-2 and the activation of caspases, thus leading to a pre-apoptotic cell behavior [103]. The caspases’ proteolytic activity in these EDS fibroblasts should produce the disassembly of actin microfilaments, as previously reported in other cell types [130]. In the absence of p125FAK, the αvβ3 integrin co-immunoprecipitates paxillin, which is distributed both in focal and fibrillar adhesions, as well as the αvβ3 integrin, talin, and vinculin. Since vinculin and paxillin are usually recruited in fibroblasts’ focal adhesion sites together with rare αvβ3 integrin patches [131], whereas fibrillar contacts consist of α5β1 integrin, tensin and talin [58,132], a different type of fibrillar adhesion is organized in cEDS and vEDS fibroblasts. Paxillin is activated trough tyrosine-phosphorylation by the p60Src kinase playing a role in EDS cells’ survival, since p60Src inhibition elicits EDS cells’ *anoikis* [103]. In these fibroblasts, paxillin is not serine-phosphorylated and does not recruit ILK, which is known to be involved in its activation [133]. The concomitant lack of ILK and p125FAK in EDS fibroblasts might contribute to the low levels of Bcl-2 expression and caspases’ activation. In addition, the αvβ3 integrin-p60Src-paxillin complexes recruit p130Cas at less extent, a docking protein that is physiologically involved in cytoskeleton remodeling [134] and that is degraded by caspases [135]. Therefore, in cEDS and vEDS fibroblasts the low amounts of p130Cas could result from the activity of caspases and could explain the actin cytoskeleton disassembly [103]. 

**Figure 2:**
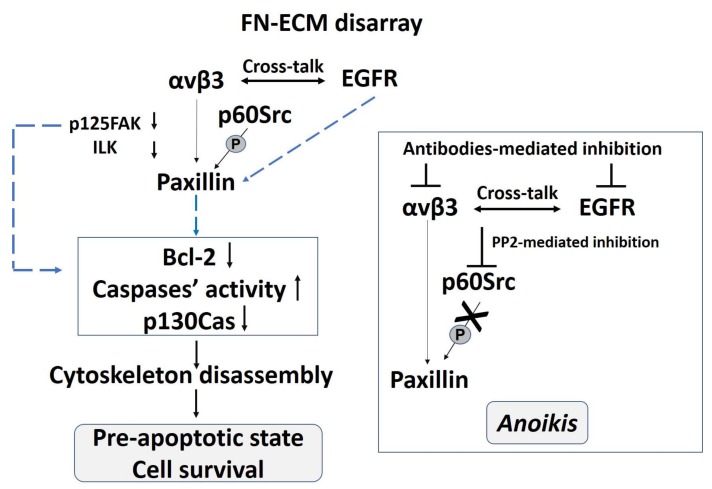
Schematic representation of the αvβ3 integrin- and EGFR-mediated signaling pathways ensuring the rescue from *anoikis* in cEDS and vEDS fibroblasts.

Furthermore, in these cells the αvβ3 integrin signaling rescues from *anoikis* by a cross-talk with EGFR, as demonstrated by the immunoprecipitation of phosphorylated EGFR with the αvβ3 integrin and by the induction of apoptosis observed either after antibody-mediated EGFR or αvβ3 inhibition or both [103], in line with several evidences that reported cross-talk mechanisms between integrins and growth factors’ receptors eliciting cell growth and rescue from apoptosis [81,82,136]. EGF–EGFR can transduce for tyrosine phosphorylation of paxillin [137], which, in turn, can act as an adaptive molecule integrating signals from integrins and growth factor receptors to ensure cell proliferation [138]. In Figure 2 a schematic representation of this survival pathway is shown.

The pre-apoptotic behavior of vEDS fibroblasts has been associated with an abnormal endoplasmic reticulum (ER) homeostasis resulting from intracellular retention of mutant and misfolded COLLIII chains. In vEDS cells, an ER perturbation was suggested by the abnormal ER distribution of the protein disulfide isomerase enzyme and by reduced expression of FKBP22, an ER resident peptidyl-prolyl *cis*-*trans*-ER isomerase involved in the folding of COLLIII triple helix [108,139,140]. Since the perturbed ER redox state may influence the down-regulation of Bcl-2 and the activation of the caspase-dependent apoptosis [141], and FKBP22 can have an anti-apoptotic role acting on the expression of Bcl-2 and caspases [142], this aspect merits further studies in vEDS fibroblasts. 

In conclusion, in cEDS and vEDS fibroblasts an in vitro survival mechanism supported by an αvβ3 integrin-EGFR cross-talk transducing to paxillin is activated, thus sustaining cell adhesion in the absence of the actin cytoskeleton. Paxillin, activated by p60Src, probably works as a strategic molecule to reinforce the αvβ3 integrin- and EGFR-mediated signaling pathways ensuring the rescue from *anoikis*.

## 3. The αvβ3 Integrin Signaling Sustains the Hypermobile EDS (hEDS) and Hypermobility Spectrum Disorders (HSD) Fibroblast-to-Myofibroblast Transition

In hEDS and HSD cells, the αvβ3 integrin was shown to be involved in the fibroblast-to-myofibroblast transition by a transduction pathway involving ILK that signals to the transcription factor Snail1 [106]. Contrary to cEDS and vEDS fibroblasts, hEDS and HSD cells exhibit a peculiar in vitro myofibroblast-like phenotype characterized by organization of the α-smooth muscle actin (α-SMA) cytoskeleton, expression of the cadherin-11, and enhanced migratory capability, probably because they synthesize high levels of MMP9, a collagenase also able to digest FN into proteolytic fragments [106]. 

**Figure 3:**
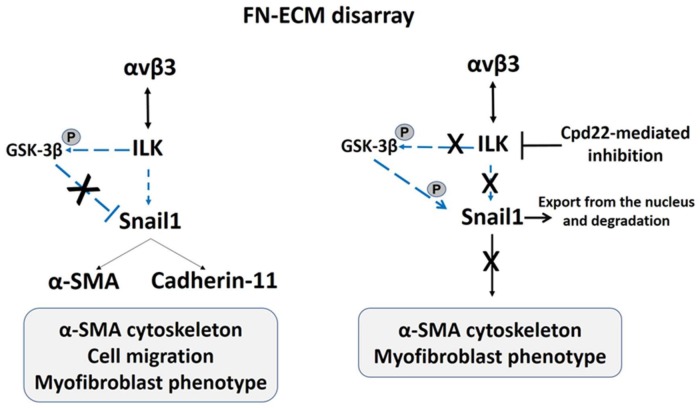
Schematic representation of the αvβ3 integrin-ILK-Snail1 transduction pathway involved in the myofibroblast-like phenotype of hEDS and HSD cells.

The implication of αv subunits-containing integrin receptors, including αvβ3, in the promotion of the α-SMA stress fibres assembly and fibroblast-to-myofibroblast transition has been investigated in different in vitro cell types [143,144]. In hEDS and HSD cells, the αvβ3 integrin binds to ILK and the β3 subunit is phosphorylated. These αvβ3 integrin-ILK complexes are recruited and activated in focal adhesion sites, promoting the α-SMA cytoskeleton’s organization, and ensuring the maintenance of the myofibroblast-like phenotype. The ILK inhibition with the liposoluble inhibitor Cpd22 induces both in hEDS and HSD cells the gradual disappearance of αvβ3 integrin and the α-SMA stress fibres disassembly [106]. Snail1, a transcription factor known to be involved in the transdifferentiation mechanisms [145], is one of the downstream effectors of the αvβ3 integrin-ILK axis in hEDS and HSD cells [106]. Indeed, it localizes both at cytoplasmic and nuclear level and immunoprecipitates either with the αvβ3 integrin or with ILK, suggesting a role in the phenotypic switch of these cells. Furthermore, the ILK inhibition delocalizes Snail1 from nuclei, confirming the activation in hEDS and HSD cells of an αvβ3 integrin-ILK-Snail1 axis sustaining the myofibroblasts’ phenotype. This condition is consistent with the role of Snail1 in the induction of myofibroblasts’ markers, i.e., cadherin-11 and α-SMA, as previously described in synovial fibroblasts from rheumatoid arthritis patients [146,147,148]. Although target molecules possibly activated by αvβ3 integrin-ILK complexes that act upstream Snail1 were not investigated in hEDS and HSD cells [106], transcriptome data in these cell models suggest a possible involvement of PI3K/Akt/GSK-3β and NF-kB signaling [107], which plays an important role in transdifferentiation mechanisms [149]. In this regard, ILK may act as kinase downstream the PI3K signaling pathway [150] and could be involved in the negative regulation, through the phosphorylation of a specific serine residue, of the glycogen synthase kinase 3β (GSK-3β) [151]. GSK-3β is a well-known kinase that physiologically controls the Snail1 export from the nucleus to the cytoplasm and its consequent degradation by ubiquitination [150,152,153]. Based on these findings, it reasonable to assume that αvβ3 integrin-ILK complexes in the focal contact sites of hEDS and HSD cells may be involved in the regulation of the GSK-3β function [106]. In hEDS and HSD cells, the ILK-mediated action should repress GSK-3β activity by its phosphorylation, thus resulting in the nuclear Snail1 expression and in the induction of the α-SMA organization. This is also sustained by the reduction of the nuclear Snail1 associated with the α-SMA disassembly observed after inhibition of the ILK kinase activity by Cpd22 in a dose-dependent manner [106], given that Cpd22 is known to prevent the ILK-mediated phosphorylation of GSK-3β [154]. In addition, ILK could phosphorylate Akt that, in turn, promotes NF-κB activation that enhances Snail1’s transcription by binding its promoter [155]. In Figure 3 a schematic representation of the αvβ3 integrin-ILK-Snail1 axis involved in the fibroblast-to-myofibroblast transition is shown.

The αvβ3 integrin’s ligands likely involved in the myofibroblast’s phenotype have not been identified yet, but the ECM disarray and, in particular, the proteolytic fragments generated by MMP9 from structural ECM components such as FN should play a key role in this signaling. Indeed, control fibroblasts, grown in the presence of the hEDS- and HSD-conditioned media, disassemble the FN–ECM and are induced to cluster at membrane level the αvβ3 integrin, which transduces through ILK to the nuclear Snail1 for the α-SMA fibers’ organization [106]. This “reprogramming” of control fibroblasts with hEDS and HSD cells’ media raises the question which factor(s), including not only growth factors and cytokines, e.g., TGF-β and Wnt, but also ECM fragments produced by proteolytic activity of different proteases such as MMP9, might be the primary contributor(s) to this transition. Indeed, it is recognized that intracellular molecules released from damaged tissues, as well as fragments of the ECM released downstream cell injury, can act as damage-associated molecular patterns (DAMPs). These molecules serve as danger signals that can elicit an immune response following tissue injury or in response to the changes in tissue composition and organization [156,157,158,159,160]. In this light, it is reasonable to assume that in hEDS/HSD cells these molecules may act as DAMPs and could be recognized by the αvβ3 integrin or other specialized receptors, which turn on pathways that transduce for the synthesis of a plethora of inflammation molecules. Both in vivo and in vitro, DAMPs could be responsible for the inflammation and chronic pain described in hEDS and HSD patients [161,162]. In this regard, hEDS and HSD cells express high levels of CCN2/CTGF, responsible for myofibroblast’ proliferation and differentiation, and low levels of CCN1/CYR61, involved in the myofibroblast’ apoptosis and resolution of inflammation [163,164]. The unbalanced synthesis of these inflammatory mediators might avoid the hEDS and HSD cells’ apoptotic death and sustain the hEDS and HSD myofibroblast-like phenotype.

Since in hEDS and HSD fibroblasts the serum deprivation does not induce cells to undergo apoptosis, the αvβ3 integrin-ILK-Snail1 pathway should be independent from serum growth factors’ action. Indeed, the contribution of mitogenic molecules in the fibroblast-to-myofibroblast transition is a thrilling topic, since myofibroblast differentiation and α-SMA expression are induced by the TGF-β [165,166]. The enhanced CCN2/CTGF expression observed in hEDS and HSD cells suggests the possible involvement of the TGF-β signaling [106]. Furthermore, it is known that CCN2/CTGF co-distributes and interacts with αv-containing integrins, including αvβ3 [167,168]. The possible involvement of the TGF-β pathway in the maintenance of the high levels of CCN2/CTGF observed hEDS ad HSD cells and, given the putative binding of CCN2/CTGF to the αvβ3 integrin, its possible role in the activation of the αvβ3 integrin-ILK-Snail1 signaling finally leading to the fibroblast-to-myofibroblast transition remain to be clarified. In this view, in different cell models increased Snail1 levels have been associated with the secretion of CCN2/CTGF, which, in turn, induces fibroblast-to-myofibroblast transition [169,170].

In conclusion, the identification of the αvβ3 integrin-ILK-Snail1 transduction pathway in hEDS and HSD cells provides insights into the molecular mechanisms likely involved in the pathophysiology of these neglected disorders. Further additional studies are needed and may represent a starting point for identifying potential therapeutic options. Quantitative protein profiling of hEDS and HSD cells’ media, aimed at clarifying which key factors secreted in their culture media are involved in the fibroblast-to-myofibroblast transition, are ongoing. In addition, the elucidation of the possible ILK-regulated downstream signaling partners, i.e., AKT and GSK-3β, which are likely involved in the transduction pathway that links the αvβ3 integrin, likely through Snail1, to the phenotypic switch of hEDS and HSD fibroblasts requires future in-depth studies.

## 4. In Arterial Tortuosity Syndrome (ATS) Dermal Fibroblasts the αvβ3 Integrin Is Involved in a Non-Canonical TGF-β Signaling 

Among the different HCTDs dermal fibroblasts shown in Table 7, only ATS fibroblasts share with EDS cells the peculiar cellular phenotype characterized by the αvβ3 integrin expression (Figure 1). In addition to the disorganization of their FN- and COLLs–ECMs, these cells also show the lack of decorin (DCN) expression and do not organize FBNs and ELN in a fibrillar network [105]. This abnormal ECM organization and particularly that of FBNs and ELN is consistent with the elastic tissue disarray reported in the arterial wall of ATS patients [98]. The exact pathomechanisms by which GLUT10 deficiency leads to the generalized ECM disarray, which reflects not only the typical ATS vascular anomalies but also the multisystem involvement overlapping with EDSs [171], were debated for long time and remain incompletely explored [98,99,105,115,116,172]. The first study that identified disease-causative variants in *SLC2A10* described an up-regulation of TGF-β signaling in arterial wall and ATS dermal fibroblasts [98]. The involvement of this pathway in the molecular pathology of ATS was sustained by the increased levels of CCN2/CTGF expression reported in ATS arterial tissue [98] and by the reduced expression of DCN, which is a TGF-β signaling inhibitor PG, in in vitro-grown patients’ fibroblasts [105]. In the last years, several evidences demonstrated that GLUT10 is localized in the ER and acts as a DHA transporter, which is the oxidized form of the ascorbic acid (AA) [99,115,116]. AA plays a major role in redox homeostasis by reducing reactive oxygen species (ROS) production, thereby protecting cells against oxidative stress. It also acts as a cofactor in reactions catalyzed by prolyl- and lysyl hydroxylases, which are a class of ER-resident enzymes involved in the maturation of COLLs and ELN [99,115,116]. ATS fibroblasts undergo oxidative stress, as shown by their high levels of ROS-mediated lipid peroxidation products [105]. However, in both the “enzyme cofactor” and “antioxidant” models of vitamin C-related pathology, the exact interaction with the TGF-β pathway remains elusive.

Since it is known that the αv-containing integrins, including αvβ3, play a key role in the activation of TGF-β [173] by interacting with the RGD motif present in the latency-associated peptide that results in the activation of the latent TGF-β [143,174,175], we hypothesized that this specific integrin might be involved in the aberrant TGF-β signaling observed in ATS fibroblasts [98,105]. Indeed, we unraveled a cross-talk mechanism between the αvβ3 integrin and a non-canonical TGF-β signaling. Specifically, in these cells the αvβ3 integrin co-immunoprecipitates and co-distributes with CCN2/CTGF, suggesting a direct interaction between αvβ3 and this growth factor, as previously reported in other cell models [168]. In ATS fibroblasts, the αvβ3 integrin transduces to p60Src through the recruitment of p125FAK, a key mediator of the TGF-β signaling [176,177], which is likely involved in the phosphorylation of the β3 integrin subunit [105]. Furthermore, ATS cells express very low levels of the TGFBRI and higher amounts of TGFBRII, which immunoprecipitates with the αvβ3 integrin. TGFBRII is tyrosine phosphorylated and p60Src should play a role in its activation, because of their co-immunoprecipitation with the αvβ3 integrin. This non-canonical pathway not only does not involve TGFBRI but also SMAD2 that is expressed at lower level than in control cells. Furthermore, TGFBRII also recruits p38MAPK that is activated by p60Src, as shown by the PP2-mediated inhibition of p60Src leading to the complete elimination of p38MAPK phosphorylation [105]. p38MAPK is a kinase involved in a wide range of signaling pathways that stimulate a multitude of different biological functions including adhesion, migration, ECM remodelling. Since p38MAPK might also be activated by stimuli other than TGF-β signaling, including an imbalance of redox homeostasis [178], it reasonable to assume that this kinase and its downstream effects play a role in the pathomechanisms of ATS. However, future investigations are needed to elucidate this intriguing aspect. 

In ATS cells, the perturbation of redox homeostasis and TGF-β signaling associated with the aberrant ECM organization is dependent on the defective GLUT10-mediated DHA transport. In fact, the stable expression of functional GLUT10 in ATS fibroblasts restores a correct DHA transport activity [99], normalizes the impaired redox homeostasis, and rescues a canonical TGF-β signaling recruiting the TGFBRI/TGFBRII receptor, which transduces to SMAD2, in the absence of αvβ3 integrin, p60Src and p38MAPK. The recovery of both redox homeostasis and canonical TGF-β signaling is associated with re-expression of DCN and, partly, reorganization of their ELN- and FBNs-ECM [105]. These observations agree with previous findings showing that the oxidative stress can induce an aberrant TGF-β signaling resulting in abnormal elastogenesis and consequent ELN disassembly into the ECM [179,180]. 

Overall, in ATS fibroblasts, GLUT10 deficiency, through a faulty intracellular DHA uptake, leads to redox unbalance and abnormal ECM protein maturation resulting in generalized ECM disorganization and activation of a non-canonical αvβ3 integrin-TGFBRII signaling, involving CCN2/CTGF, p125FAK, p60Src, and p38MAPK. 

## 5. Concluding Remarks

Human dermal fibroblasts from healthy individuals organize in vitro several structural ECM components that recruit specific integrin receptors. FN is assembled in a fibrillar network through the binding with the α5β1 integrin. In the majority of EDS and in ATS fibroblasts carrying mutations in different ECM-related genes, but not in those derived from LDS, MFS and OI patients, the FN–ECM and the α5β1 integrin are disorganized and strongly reduced respectively and, consequently, the alternative FN receptor the αvβ3 integrin, which is almost absent in control fibroblasts, is recruited. The different implications of the αvβ3 integrin identified in cultured cEDS, vEDS, hEDS, HSD, and ATS dermal fibroblasts highlight the multifaced nature of this integrin at least in this in vitro cell model. In cEDS and vEDS cells, the αvβ3 integrin exerts a pro-survival role promoting cell adhesion and preventing *anoikis* through p60Src-paxillin-mediated signaling and EGFR cross-talking. In hEDS and HSD cells, the αvβ3 integrin is involved in the fibroblast-to-myofibroblast transition through the interaction with ILK that signals to the transcription factor Snail1. In ATS fibroblasts, the αvβ3 integrin cross-talks with a TGFBRII-mediated non-canonical TGFβ signaling. 

In conclusion, these data show that αvβ3 integrin, specifically recruited by EDS and ATS cells, participates in different signaling pathways depending on the specific disorder and underlying molecular defects. The αvβ3 integrin is sufficiently multifaced to switch on different signaling cascades downstream of the interplay enrolled with its numerous intracellular interactors and by its cross-talk capability with different growth factor-receptor complexes. These findings add insights into the manifold functions of the αvβ3 integrin, but more effort is required to deepen knowledge on the numerous partners identified in the reported pathways that could shed light on pathomechanisms associated with these HCTDs. In addition, further functional studies are also needed to ascertain whether similar or different αvβ3-mediated signaling mechanisms are elicited in the other EDS fibroblasts expressing this integrin.

## Figures and Tables

**Table 1:** Cell expression, major ligands, and main functions of the αvβ3 integrin.

**Cell types**	Endothelial cells, smooth muscle cells, myofibroblasts, osteoclasts, monocytes, platelets, fibroblasts, tumor cells (melanoma, glioblastoma, pancreatic, prostate, ovarian, breast tumor cells), placenta
**Ligands**	Vitronectin, fibrinogen, von Willebrand factor, thrombospondin, prothrombin, fibronectin, fibrillins, laminin, osteopontin, bone sialoprotein
**Functions**	Cell adhesion, cell migration, cell survival and proliferation, growth factor deprivation-induced apoptosis rescue, *anoikis* rescue, angiogenesis, hemostasis, platelet aggregation, wound healing, fibrosis, inflammation, tumor cells’ invasion and metastasis, restenosis, bone resorption, activation of latent TGF-β, embryonic development

**Table 2:** Ehlers–Danlos syndromes (EDS) types according to the 2017 revised nosology.

EDS Type	IP	Gene	Protein
Classical EDS (cEDS)	AD	Major: *COL5A1*, *COL5A2* Rare: *COL1A1*	Type V collagen Type I collagen
Classical-like EDS (clEDS)	AR	*TNXB*	Tenascin-X
Cardiac-valvular EDS (cvEDS)	AR	*COL1A2*	Type I collagen
Vascular EDS (vEDS)	AD	*COL3A1*	Type III collagen
Hypermobile EDS (hEDS)	AD	Unknown	Unknown
Arthrochalasia EDS (aEDS)	AD	*COL1A1*, *COL1A2*	Type I collagen
Dermatosparaxis EDS (dEDS)	AR	*ADAMTS2*	ADAMTS-2
Kyphoscoliotic EDS (kEDS)	AR	*PLOD1* *FKBP14*	LH1 FKBP22
Brittle cornea syndrome (BCS)	AR	*ZNF469* *PRDM5*	ZNF469 PRDM5
Spondylodysplastic EDS (spEDS)	AR	*B4GALT7* *B3GALT6* *SLC39A13*	β4GalT7 β3GalT6 ZIP13
Musculocontractural EDS (mcEDS)	AR	*CHST14* *DSE*	D4ST1 DSE
Myopathic EDS (mEDS)	AD/AR	*COL12A1*	Type XII collagen
Periodontal EDS (pEDS)	AD	*C1R* *C1S*	C1r C1s

IP: inheritance pattern; AD: autosomal dominant; AR: autosomal recessive.

**Table 3:** Clinical criteria for classical Ehlers-Danlos syndrome (cEDS) according to the revised 2017 EDS nosology.

Major Criteria	Minor Criteria
1. Skin hyperextensibility and atrophic scarring 2. Generalized joint hypermobility (BS ≥ 5)	1. Easy bruising 2. Soft, doughy skin 3. Skin fragility (or traumatic splitting) 4. Molluscoid pseudotumors 5. Subcutaneous spheroids 6. Hernia (or history thereof) 7. Epicanthal folds 8. Complications of joint hypermobility (e.g., sprains, luxation/subluxation, pain, flexible flatfoot) 9. Family history of a first degree relative who meets clinical criteria
**Minimal criteria suggestive for cEDS:** Major criterion (1) Plus either: major criterion (2) and/or: at least three minor criteria.	

**Table 4:** Clinical criteria for vascular Ehlers-Danlos syndrome (vEDS) according to the revised 2017 EDS nosology.

Major Criteria	Minor Criteria
1. Family history with documented *COL3A1* variant 2. Arterial rupture at a young age 3. Spontaneous sigmoid colon perforation 4. Uterine rupture during the third trimester in the absence of previous C-section and/or severe peripartum perineum tears 5. Carotid-cavernous sinus fistula formation in the absence of trauma	1. Bruising unrelated to identified trauma 2. Thin, translucent skin with increased venous visibility 3. Characteristic facial appearance 4. Spontaneous pneumothorax 5. Acrogeria 6. Talipes equinovarus 7. Congenital hip dislocation 8. Hypermobility of small joints 9. Tendon and muscle rupture 10. Keratoconus 11. Gingival recession/fragility 12. Early-onset varicose veins
**Minimal criteria suggestive for vEDS:** Family history of the disorder, arterial rupture or dissection in individuals less than 40 years of age, unexplained sigmoid colon rupture, or spontaneous pneumothorax in the presence of other features consistent with vEDS.	

**Table 5:** Clinical criteria for hypermobile Ehlers-Danlos syndrome (hEDS) according to the revised 2017 EDS nosology.

The Clinical Diagnosis of hEDS Needs the Simultaneous Presence of Criteria 1 and 2 and 3		
**Criterion 1**	**Criterion 2**	**Criterion 3**
	Two or more among the features A–C must be present	All must be met
1. Generalized joint hypermobility: BS ≥ 6 for pre-pubertal children and adolescents; BS ≥ 5 for pubertal men and women up to the age of 50; BS ≥ 4 for those >50 years of age	**SIGN A** (a total of five must be present): 1. Unusually soft or velvety skin 2. Mild skin hyperextensibility 3. Unexplained striae 4. Bilateral piezogenic papules of the heel 5. Recurrent or multiple abdominal hernia(s) (e.g., umbilical, inguinal, crural) 6. Atrophic scarring involving at least two sites and without the formation of truly papyraceous and/or hemosiderotic scars 7. Pelvic floor, rectal, and/or uterine prolapse in children, men or nulliparous women 8. Dental crowding and high or narrow palate 9. Arachnodactyly 10. Arm span-to-height ≥ 1.05 11. Mitral valve prolapse mild or greater based on strict echocardiographic criteria 12. Aortic root dilatation with Z-score > +2 **SIGN B:** Positive family history, with one or more first degree relatives independently meeting the diagnostic criteria for hEDS **SIGN C** (at least one): 1. Musculoskeletal pain in two or more limbs, recurring daily for at least 3 months 2. Chronic, widespread pain for ≥3 months 3. Recurrent joint dislocations or frank joint instability, in the absence of trauma	1. Absence of unusual skin fragility, which should prompt consideration of other types of EDS 2. Exclusion of other HCTDs, including autoimmune and rheumatologic conditions 3. Exclusion of alternative diagnoses that include joint hypermobility by means of hypotonia and/or connective tissue laxity

**Table 6:** Overview of the clinical features of arterial tortuosity syndrome (ATS).

**Craniofacial:** Aged appearance; long face; hypertelorism; downslanting palpebral fissures; beaked nose; cleft palate/bifid uvula; high arched palate; micrognathia; sagging cheeks
**Ocular:** Keratoconus; keratoglobus; myopia
**Cutaneous:** Velvety texture; thin skin; hyperextensible skin; cutis laxa
**Skeletal:** Pectus deformity; scoliosis; arachnodactyly; joint hypermobility and pain
**Cardiovascular:** Aortic tortuosity; tortuosity of other arteries; abnormal implantation of the aortic branches; aortic root aneurysm; other arterial aneurysms; arterial dissections; stenosis of the pulmonary arteries; aortic stenosis
**Other manifestations:** Diaphragmatic hernia; inguinal hernia; respiratory symptoms; urogenital abnormalities; autonomic dysfunction

**Table 7:** Organization of FN and COLLs and their canonical integrin receptors in control, EDS, and other HCTDs dermal fibroblasts.

ECM Components and Integrins #	Control Fibroblasts	cEDS *COL5A1* *COL5A2*	vEDS *COL3A1*	hEDS HSD Unknown	kEDS *FKBP14*	kEDS ^u^ *PLOD1*	dEDS ^u^ *ADAMTS2*	aEDS ^u^ *COL1A2* ex6	BCS *PRDM5* *ZNF469*	mcEDS *CSHT14*	spEDS *B3GALT6*	LDS ^u^ *TGFBR1*	MFS ^u^ *FBN1*	OI ^u^ *COL1A1* *COL1A2*	ATS *SLC2A10*
**FN**	++	+	+	+	+	+	+	+	+	+	++	++	++	++	+
**α5β1**	**	*/-	*/-	*/-	-	-	-	-	*/-	-	**	**	**	**	*^,u^
**αvβ3**	-	**	**	**	**^,u^	**	**	**	**^,u^	**^,u^	-^,u^	-	-	-	**
**COLLI**	+	+/-	-	-	-	-	-	-	-	-	+	+	+	-	-
**COLLIII**	++	-	-	-	-	-	-	-	-	-	-	-	+	-	-
**COLLV**	++	-	++/+	-	++	-	-	-	+	+	+	++	++	+/-	+/-
**α2β1**	**	-	-	-	-	-	-	-	-	-	-	na	*	-	-^,u^

#: detected by immunofluorescence analyses, ++: abundant and fibrillar ECM, +: rare ECM fibrils, **: abundant patches in plasma membrane, *: reduced patches in plasma membrane, -: negligible amounts/absent, na: not analyzed, u: unpublished. The genes underlying the different HCTDs are reported in italics.

## Glossary

**Extracellular matrix (ECM)**	ECM is a three-dimensional structure that encapsulates cells and defines their microenvironment, providing a physical scaffolding for the cellular constituents. ECM is a dynamic structure, constantly undergoing a remodeling process whereby ECM components are deposited, degraded, or modified. ECM dynamics are essential during restructuring of tissue architecture.
**Integrins**	A family of cell adhesion receptors that mediate either cell–cell interactions or cell–ECM interactions. Integrins are heterodimers with two distinct subunits, the α-subunit and the β-subunit.
**Fibroblasts**	The major cells responsible for the production of collagens, glycosaminoglycans, and proteoglycans, which are major components of the ECM.
**Myofibroblasts**	Specialized cells with both fibroblasts’ and SMCs’ phenotypic characteristics. These cells are activated by inflammatory cytokines and are involved in wound-healing mechanisms and in pathological conditions, such as fibrosis and chronic inflammation. They peculiarly express the α-SMA that they organize into the cytoskeleton to generate contractile force and migrate.
**Fibroblast-to-myofibroblast transition**	Phenotypic conversion of fibroblast into myofibroblast by transdifferentiation mechanisms occurring during wound healing, fibrosis, and inflammation. A well-characterized hallmark of the fibroblast-to myofibroblast transition is *the novo* formation of α-SMA stress fibers.
**Focal adhesions**	Transmembrane anchorage sites organized on the lower surface of the cell and in the cell’s periphery to anchor the underlying ECM fibrils. These structures are associated with the end of actin stress fibers and usually contain the αvβ3 integrin that interacts with a complex pattern of proteins including vinculin, talin, paxillin, α-actinin, zyxin, p125FAK, ILK, and other phosphotyrosine proteins and kinases.
**Fibrillar adhesions**	Integrin-containing complexes arising from focal adhesions, distributed on the upper cell surface, involved in the ECM fibrils’ organization. These elongated structures are enriched in α5β1 integrins bound to tensin.
***Anoikis***	Mechanism of programmed cell death or apoptosis induced by the loss of cell-ECM adhesion. Consequently, the ECM can be considered a survival signal for cells.
**Cross-talk mechanism**	Mechanism by which two or more surface receptors or two or more interactors recruited in different signal-transduction pathways affect each other and reinforce the downstream cell response to an extracellular signal.
**Damage-associated molecular patterns (DAMPs)**	Molecules released from damaged tissues, such as components or fragments of the ECM, released downstream of the cell injury. These danger signals bind specific receptors, such as Toll-like receptors, to elicit an immune response following tissue injury or in response to the changes in tissue composition and organization. DAMPs can also play a role in chronic pain conditions.
**Tissue homeostasis**	A homeostatic process involved in the maintenance of an internal steady state within a defined tissue of an organism, including control of cellular proliferation and death and control of metabolic function.
**Redox homeostasis**	Balance between intracellular reactive oxygen species (ROS) generation and elimination.
